# Investigation of Efficient Pullulan Synthesis Utilizing Huangjiu Lees as a Substrate

**DOI:** 10.3390/foods13233874

**Published:** 2024-11-29

**Authors:** Peiqi Lu, Tiantian Liu, Jingqiu Ma, Tao Kan, Xiao Han, Zhongwei Ji, Jian Mao

**Affiliations:** 1 School of Food Science and Technology, National Engineering Research Center of Cereal Fermentation and Food Biomanufacturing, Jiangnan University, Wuxi 214122, China; 6230111208@stu.jiangnan.edu.cn (P.L.); liutiantain@jiangnan.edu.cn (T.L.); hanxiao@jiangnan.edu.cn (X.H.); jizhongwei@jiangnan.edu.cn (Z.J.); 2 Jiangnan University (Shaoxing) Industrial Technology Research Institute, Shaoxing 312000, China; majingqiu1224@163.com (J.M.); kantao0610@163.com (T.K.)

**Keywords:** *Aureobasidium pullulans*, pullulan, huangjiu lees, resource utilization, transcriptome analysis

## Abstract

Pullulan is a high-value biopolymer synthesized by *Aureobasidium pullulans* through the fermentation of starch and sugars. It finds extensive applications in food, packaging, biomedicine, and other sectors. However, the high production costs significantly limit the development and application of pullulan. Consequently, there is an urgent need to identify high-quality fermentation substrates. In recent years, the rapid growth of Huangjiu industry has led to the generation of waste Huangjiu lees, which not only contribute to environmental pollution but also represent a significant waste of resources. As a result, the resource utilization of Huangjiu lees has garnered considerable attention. In this study, Huangjiu lees were employed as raw materials for fermentation to produce pullulan. Following fermentation of Huangjiu lees powder with the primary strain *Aureobasidium pullulans* LL1, the yield of pullulan was notably reduced. Through adaptive evolution, an evolved strain, *Aureobasidium pullulans* AP9, was isolated, demonstrating enhanced efficiency in producing pullulan from Huangjiu lees. The impact of Huangjiu lees on pullulan biosynthesis was elucidated via transcriptome analysis. Fermentation conditions were optimized using a single-factor approach, and a multi-strain staged fermentation strategy involving *Aspergillus niger* and *Aureobasidium pullulans* was employed to further enhance pullulan yield. Under optimal conditions, the pullulan yield reached 22.06 g/L, with a molecular weight of 1.04 × 10^6^ Da. This study underscores the significant potential of utilizing Huangjiu lees for pullulan production and offers valuable insights for the resource utilization of this byproduct.

## 1. Introduction

Huangjiu lees are the solid byproducts obtained from the pressing of fermented Huangjiu mash [1]. Research indicates that the main components of Huangjiu lees include starch, proteins, amino acids, and cellulose, which possess significant potential for reuse [2]. With the annual increase in the production and consumption of Huangjiu, the output of Huangjiu lees is also on the rise [3]. Consequently, the resource utilization of Huangjiu lees has become an issue that cannot be overlooked and requires urgent attention. However, the current utilization methods for Huangjiu lees are relatively simplistic, with most being used as animal feed or biomass fuel, resulting in low-value applications, while the rich nutrients contained within are not efficiently converted [4,5,6]. Currently, there is limited research on the microbial utilization of Huangjiu lees, despite the fact that the abundant nutrients present are essential for microbial cultivation. Utilizing microorganisms to ferment Huangjiu lees as a substrate for production represents an effective approach to achieving the resource utilization of Huangjiu lees [7].

*Aureobasidium pullulans* (*A. pullulans*) is a polymorphic fungus with an exceptionally complex life cycle, typically existing in five morphological forms: yeast-like, conidiospores, swollen spores, thick-walled spores, and mycelium [8]. This organism can utilize various carbohydrates, glycerol, pectin, and tannic acid as carbon sources to efficiently synthesize pullulan, while also producing economically valuable melanin [9,10,11,12,13]. Pullulan is an extracellular water-soluble mucilaginous polysaccharide similar to glucan, and it is one of the main fermentation products of *A. pullulans* [14]. Studies indicate that the chemical formula of pullulan is (C_6_H_10_O_5_)n, with a molecular weight typically ranging from 20,000 to 2,000,000. This polysaccharide is primarily composed of maltotriose repeating units linked by α-1,4-glycosidic bonds, which are further polymerized into a linear polysaccharide through α-1,6-glycosidic bonds [15]. Due to its unique structure and excellent properties, pullulan exhibits broad application prospects in food processing, pharmaceutical, and chemical industries [16].

Currently, the industrial production of pullulan typically utilizes sucrose and glucose as carbon sources, with yeast extract serving as a nitrogen source, and employs *A. pullulans* for fermentation [17]. However, these essential nutrients account for a significant portion of the production costs of pullulan, thereby limiting its large-scale production [18]. Consequently, reducing the industrial production costs of pullulan and developing production methods using inexpensive non-food biomass [19,20,21] are crucial for enhancing its competitiveness against other microbial polysaccharides. There have been numerous studies on the production of pullulan from non-food biomass using *A. pullulans*. Wang et al. [18] isolated a strain of *A. pullulans* capable of fermenting rice husk hydrolysate to produce pullulan. Tagne et al. [22] cultivated *A. pullulans* in a batch reactor using sugarcane bagasse as the raw material. Zhang et al. [23] investigated the production of pullulan using potato residue as the substrate with *A. pullulans*. These studies demonstrate the strong environmental adaptability of *A. pullulans*, which can utilize various non-food biomass sources for pullulan production [24]. However, the regulatory pathways and adaptive strategies involved in the polysaccharide production response of *A. pullulans* remain to be fully understood.

This study uses Huangjiu lees as a substrate to investigate its effect on the production of pullulan by *A. pullulans*. Through adaptive evolution, an evolved strain of *A. pullulans* capable of efficiently utilizing Huangjiu lees for pullulan production was obtained. Subsequently, transcriptomic analysis was conducted to explore the gene expression patterns during the fermentation of Huangjiu lees by *A. pullulans*, along with differential gene expression analysis. Finally, the fermentation process of the *A. pullulans* strain was optimized to achieve efficient production of pullulan using Huangjiu lees. This research not only provides support and insights for the industrial production of pullulan but also offers valuable guidance for the resource utilization of Huangjiu lees.

## 2. Materials and Methods

### 2.1. Strains and Culture Media

*A. pullulans* LL1 and *Aspergillus niger* used in this study were isolated and preserved by our laboratory. The composition of the seed culture medium includes the following: sucrose, 20 g/L; yeast extract, 2.5 g/L; (NH_4_)_2_SO_4_, 0.6 g/L; K_2_HPO_4_, 5.0 g/L; NaCl, 1.0 g/L; and MgSO_4_, 0.2 g/L. The fermentation medium consists of the following: sucrose, 50 g/L; yeast extract, 2.5 g/L; (NH_4_)_2_SO_4_, 0.6 g/L; K_2_HPO_4_, 5.0 g/L; NaCl, 1.0 g/L; and MgSO_4_, 0.2 g/L. For the Huangjiu lees medium, sucrose in the fermentation medium is replaced with 50 g/L of Huangjiu lees powder; for the Huangjiu lees hydrolysate medium, sucrose is replaced with 50 mL/L of Huangjiu hydrolysate lees [25].

### 2.2. Pre-Treatment of Raw Materials for Huangjiu Lees

Preparation of Huangjiu lees powder: Fresh Huangjiu lees was dried in an oven at 55 °C and then ground using a pulverizer to obtain Huangjiu lees powder for later use [25].

Preparation of Huangjiu lees hydrolysate: Huangjiu lees powder was pre-mixed with a 2% mixed acid solution (1% sulfuric acid and 1% phosphoric acid) at a concentration of 10% (*w*/*v*). The mixture was reacted at 120 °C for 3 h, followed by 30 min of ultrasonic treatment. After neutralization with sodium hydroxide solution, solid residues were separated to obtain the filtrate. The filtrate was decolorized using activated carbon to produce Huangjiu lees hydrolysate [26].

### 2.3. Cultivation of A. pullulans

A ring of *A. pullulans* LL1 was selected from the glycerol tube and inoculated onto solid plates for activation. It was incubated at 26 °C for 96 h, after which individual colonies were picked and transferred to a liquid seed culture medium, maintaining a pH of 6.5. This was then incubated at 26 °C on a shaking incubator at 160 rpm for 72 h to obtain the primary seed liquid. Subsequently, a 5% inoculation rate was applied, and the primary seed liquid was introduced into fermentation media, media supplemented with Huangjiu lees powder, and media containing hydrolyzed Huangjiu lees, all incubated at 26 °C on a shaking incubator at 180 rpm for 192 h [27].

### 2.4. Determination of the Yield and Molecular Weight of Pullulan

The fermentation broth was centrifuged at 10,000 rpm for 10 min at 4 °C. The supernatant was collected and precipitated with an equal volume of absolute ethanol and then stored at 4 °C for 24 h. Subsequently, the precipitate was collected using centrifugation at 10,000 rpm at 4 °C and washed twice with absolute ethanol. It was then dried in an oven at 65 °C until a constant weight was achieved, yielding pullulan. Pullulan was dissolved in deionized water to prepare a polysaccharide solution at a concentration of 0.1 g/L. The viscosity of the polysaccharide solution was measured at 25 °C using a Ubbelohde capillary viscometer (ZhenJing glass instrument factory, Zhenjiang, China). The molecular weight of pullulan was calculated using the Huggins equation (η = 0.000258 × Mw^0.646^) [27].

### 2.5. Adaptive Evolution

The adaptive evolution of *A. pullulans* LL1 was conducted by sequentially transferring it to a selective medium containing a high concentration of Huangjiu lees. The selective medium refers to a culture medium in which sucrose is gradually replaced by Huangjiu lees, with the proportion of Huangjiu lees increasing from 0% to 100% during the subculturing process, ultimately resulting in the complete replacement of sucrose. The evolved strain was cultured for 10 generations to study colony stability and the yield of pullulan [27].

### 2.6. Transcriptome Sequencing and Analysis

According to the method described in Section 2.3, the original strains and evolved strains were inoculated into Huangjiu lees medium and cultured at 28 °C with shaking at 180 rpm for 72 h. Total RNA was extracted from the samples, after which rRNA was removed using a conventional kit to isolate mRNA, which was then enriched using mRNA Capture Beads. Following purification with the beads, the mRNA was fragmented using high temperature. The fragmented mRNA served as a template for synthesizing the first strand of cDNA in a reverse-transcriptase reaction mixture. Simultaneously, the second strand of cDNA was synthesized, along with end repair and the addition of an A tail. Subsequently, adapters were ligated, and target fragments were purified using Hieff NGS^®^ DNA Selection Beads. After PCR amplification and quality assessment, sequencing was performed on the Illumina platform.

RNA library sequencing was performed on the Illumina NovaSeq X Plus by Gene Denovo Biotechnology Co., Ltd. (Guangzhou, China).

### 2.7. Fermentation Process Optimization

Using Huangjiu lees medium and the evolved strain AP9 of *A. pullulans*, a 5 L fermentation tank experiment was conducted to explore the optimal production conditions. Single-factor conditions such as fermentation time, initial pH, inoculum quantity, aeration rate, rotational speed, and temperature were selected as variables, with the pullulan yield and molecular weight serving as evaluation indicators for the experiments. The basic parameters were as follows: a fermentation time of 5 d, an initial pH of 6.0, an inoculation quantity of 1.0%, an aeration rate of 0.5 vvm, a rotational speed of 250 rpm, and a temperature of 26 °C. Specific parameters are detailed in Table 1.

A segmented fermentation method was employed to study the high-yield production of pullulan using Huangjiu lees. First, *Aspergillus niger* was inoculated into the Huangjiu lees medium, followed by sterilization, after which the *A. pullulans* LL1 was introduced. The inoculation amount and timing of *Aspergillus niger* were selected as influencing factors, with pullulan yield and molecular weight serving as the indicators for the fermentation experiments. Specific parameters are detailed in Table 2.

### 2.8. Primer Design and Gene Expression Verification

Using the method described in Section 2.3, total RNA was extracted from the primary strain of *A. pullulans* LL1 and the evolved strain AP9 using TRIzol reagent (Meryer Biochemical Technology Co., Ltd., Shanghai, China). To verify the accuracy of the transcriptome sequencing results, five differentially expressed genes (AmyA, ags1, bglA, MFS, and SGT) were selected for RT-qPCR experiments. Supplementary Table S1 lists the primers used for RT-qPCR to detect gene expression, and the 2^−ΔΔCT^ method was employed to assess gene expression levels.

### 2.9. Statistical Analysis

Data from three independent experiment were averaged and expressed as means ± standard deviation. All data were plotted using Origin Pro 2022 software (OriginLab Corporation, Northampton, MA, USA). One-way ANOVA was conducted using SPSS 26.0 software (IBMCorp., Armonk, NY, USA), with *p* < 0.05 typically considered statistically significant.

## 3. Results and Discussion

### 3.1. Effects of Different Pretreatment Methods of Huangjiu Lees on Pullulan Biosynthesis

In the process of Huangjiu brewing, Huangjiu lees are primarily obtained through pressing, resulting in a dense structure. If untreated Huangjiu lees are used, this density cannot make the microorganisms make good use of the nutrients in it, and there are a lot of inhibiting substances such as alcohol in untreated Huangjiu lees, which affects the normal cell metabolism of *A. pullulans* [28]. Consequently, effective resource utilization of Huangjiu lees necessitates a pretreatment to disrupt their compact structure, thereby facilitating the release of nutrients and reducing or eliminating inhibitory factors. This study employed both physical and chemical methods to pretreat Huangjiu lees. Subsequently, *A. pullulans* LL1 was cultivated using Huangjiu lees powder and hydrolyzate as raw materials. The effects of various pretreatment methods on pullulan synthesis were also investigated.

**Figure 1:**
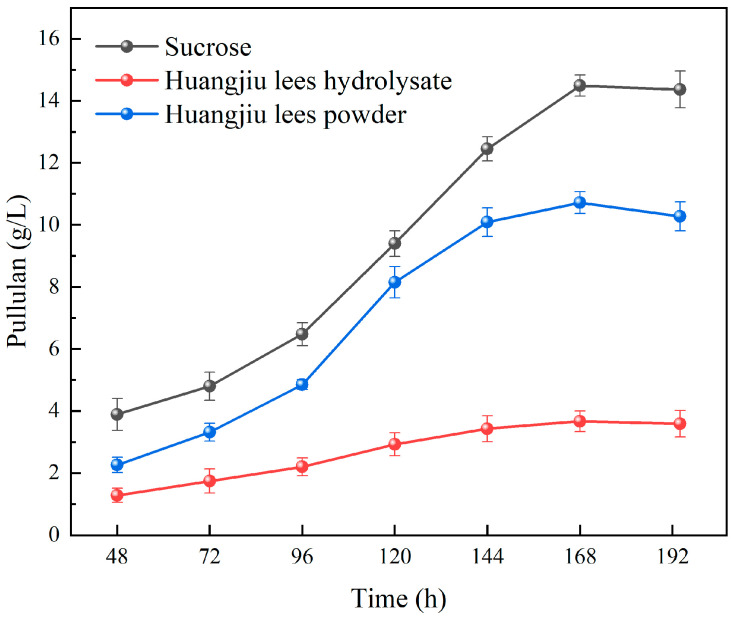
Effects of sucrose, Huangjiu lees powder, and Huangjiu lees hydrolyzate on pullulan production by *A. pullulans* LL1.

As illustrated in Figure 1, the fermentation of pullulan by *A. pullulans* LL1 using different substrates—sucrose, Huangjiu lees powder, and Huangjiu lees hydrolysate—results in significantly varying yields of pullulan. Previous studies have identified sucrose as the optimal carbon source for pullulan fermentation by *A. pullulans* [28]. The findings from this experiment corroborate that the fermentation efficacy of Huangjiu lees powder and hydrolysate is inferior to that of sucrose. Specifically, the pullulan yields from these two substrates are markedly lower than those from sucrose, suggesting that Huangjiu lees disrupt the normal metabolic processes of *A. pullulans* LL1. This disruption may be attributed to the inability of both pretreatment methods to effectively eliminate inhibitory factors, rendering *A. pullulans* LL1 highly sensitive to Huangjiu lees and hindering its growth and fermentation capabilities, which ultimately leads to reduced pullulan yield. A comparative analysis of the effects of different pretreatment methods for Huangjiu lees on pullulan yield reveals that the yield when using Huangjiu lees hydrolysate as a substrate is significantly lower than that obtained with Huangjiu lees powder. This indicates that the pretreatment via acid hydrolysis of Huangjiu lees is ineffective, rendering it unsuitable for pullulan production. Consequently, subsequent studies were conducted using Huangjiu lees powder as the substrate. Given the aforementioned results, it is evident that the affinity between the primary strain and Huangjiu lees is insufficient. Therefore, it is imperative to perform laboratory adaptive evolution experiments on the primary strain, *A. pullulans* LL1, to develop an evolved strain capable of adapting to the components of Huangjiu lees and efficiently utilizing them for pullulan production.

### 3.2. Adaptive Evolution of A. pullulans

Adaptive evolution technology typically relies on specific or progressively imposed selection pressures to identify strain mutants with superior performance. This approach is particularly effective in enhancing the stress resistance of microbial strains, making it a common method for improving strain performance under varied production conditions [29]. In this study, Huangjiu lees powder was added to the fermentation medium, with its concentration gradually increased until sucrose was completely substituted. Adaptive evolution experiments were conducted on the primary strain, *A. pullulans* LL1. After ten passages, a strain exhibiting improved growth was isolated and designated *A. pullulans* AP9. The original strain exhibited a limited ability to utilize Huangjiu lees powder for fermentation, resulting in low yield and molecular weight of pullulan. However, with the progression of adaptive evolution, the affinity of *A. pullulans* for Huangjiu lees increased. Consequently, the yield and molecular weight of pullulan from the evolved strain significantly improved, indicating that adaptive evolution enhances the ability of *A. pullulans* to effectively utilize Huangjiu lees powder, thereby maximizing pullulan yield.

**Figure 2:**
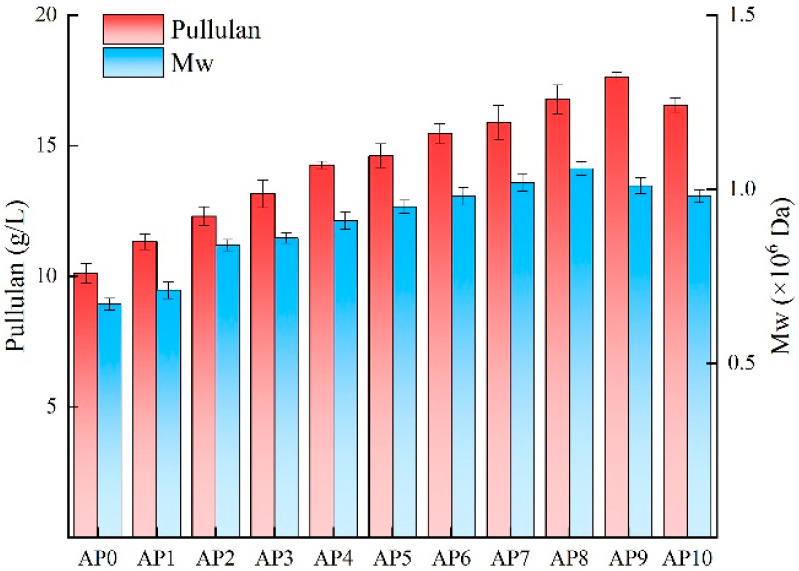
Pullulan yield and molecular weight of different strains of *A. pullulans* in shake flask culture using Huangjiu lees medium.

Research indicates that *A. pullulans* has a strong capacity for environmental adaptation, allowing it to evolve under adverse conditions and efficiently utilize various substrates for growth and metabolic activities [30]. To demonstrate the ability of evolved strains to adapt to the components of Huangjiu lees for the efficient production of pullulan, experiments were conducted using the original strain and each generation of evolved strains in media containing the same concentration of Huangjiu lees powder. The results, depicted in Figure 2, show that each generation of evolved *A. pullulans* strains grew well and produced pullulan. However, due to varying adaptability to Huangjiu lees among the strains, there were significant differences in pullulan yield and molecular weight across generations. Under the same concentration of Huangjiu wine lees powder, the pullulan yield of each generation gradually increased, with AP9 achieving the highest yield, which was 74.28% greater than that of the original strain. This indicates that the adaptability of strains improved from AP1 to AP9, with AP9 exhibiting the best adaptation to Huangjiu lees and the strongest pullulan synthesis capability. Thus, adaptive evolution can be effectively applied in the domestication of microorganisms for practical production, enhancing the yield of target products and increasing their added value. AP9 is an ideal strain for the efficient synthesis of pullulan and holds significant application potential in the resource utilization of Huangjiu lees. The essence of adaptive evolution lies in enhancing the expression levels of key genes involved in product synthesis; therefore, subsequent transcriptome sequencing of the original and evolved strains will reveal the reasons for the differences in pullulan yield from the perspective of gene expression.

### 3.3. Transcriptome Sequencing Gene Statistics and Sample Relationships

The sequencing results indicate that the reference genome contains 14,099 genes, with a total of 13,482 genes detected during the sequencing process, accounting for 95.62% of the reference genome. Specifically, the primary AP0 group detected 13,088 genes, representing 92.83%, while the evolved AP9 group detected 12,887 genes, corresponding to 91.40%. Detailed statistics on the number of detected genes for each sample can be found in Supplementary Table S2. These results support the validity of the selected reference genome and its applicability for subsequent differential gene expression analysis.

**Figure 3:**
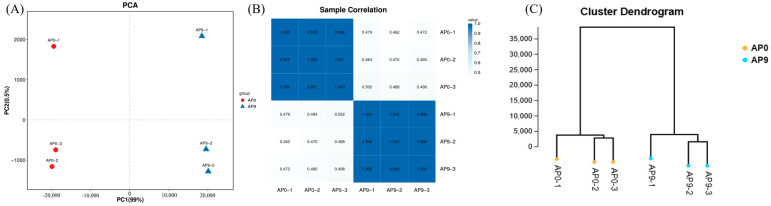
Sample relationship. (**A**) Principal component analysis diagram; (**B**) correlation heat map; (**C**) sample clustering diagram.

Based on the expression levels of known genes in each sample, principal component analysis (PCA) and Pearson correlation coefficient analysis were employed to assess the reproducibility among samples, facilitating the identification and exclusion of outliers. Utilizing expression data, we conducted PCA in R to investigate the distance relationships among samples, reflecting the complex composition of samples in two feature values on the x and y coordinates, thereby uncovering clustering patterns within the complex data. The analysis indicated that samples with similar compositions were closer together in the PCA plot, while samples from different effective treatments typically exhibited distinct clustering distributions. A two-dimensional coordinate plot, as shown in Figure 3A, was generated, where the PC1 coordinate represents the first principal component, and the percentage in parentheses indicates its contribution to the variance among samples; the PC2 coordinate represents the second principal component, with the percentage in parentheses denoting its contribution to the variance. Colored points in the figure represent individual samples. The analysis revealed minimal differences between groups and a high degree of similarity among samples. PCA provides a straightforward method for visualizing relationships between samples, while correlation coefficients offer a more detailed assessment of the strength of these relationships. By calculating the Pearson correlation coefficients for expression levels between any two samples, we visually represented these correlations in a heatmap format, allowing for the evaluation of reproducibility among repeated samples within groups. The results are presented in Figure 3B, where both axes correspond to individual samples, and the color intensity indicates the magnitude of the correlation coefficients—darker shades of blue signify stronger correlations, while lighter shades indicate weaker correlations—thereby aiding in the identification of differentially expressed genes. The sample clustering diagram, derived from the expression levels of all genes or a target gene set, presents the results of hierarchical clustering of all samples to reflect their relationships, assisting in the assessment of sample reproducibility and the detection of outliers. The sample clustering diagram in Figure 3C indicates that samples from the AP0 group and AP9 group formed distinct clusters without any outliers, suggesting a significant difference between the AP0 and AP9 groups. These findings are consistent with previous experimental results regarding the pullulan yield and molecular weight.

### 3.4. Gene Expression Analysis

**Figure 4:**
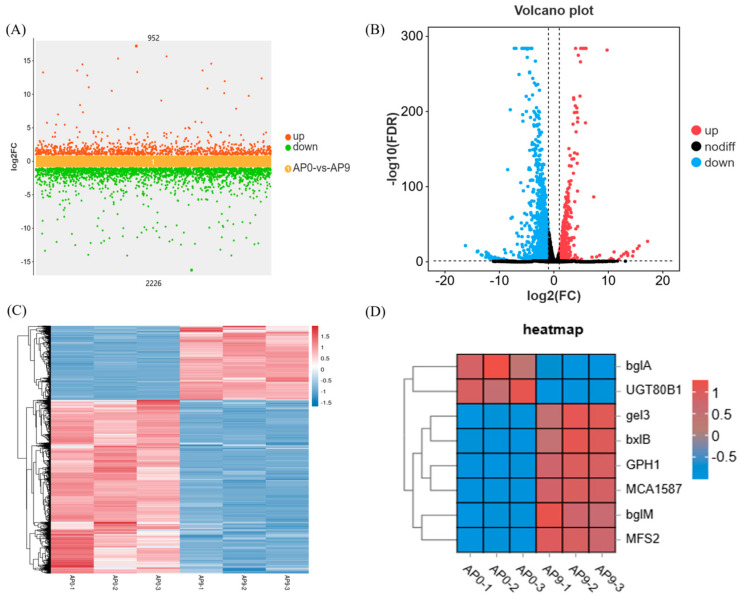
Gene expression analysis. (**A**) Multi-group difference scatter plot; (**B**) AP0 and AP9 differential gene volcano plot; (**C**) differential gene cluster heat map; (**D**) pullulan synthesis related gene expression heat map.

Multiple sets of differential scatter plots use the logarithmic value of the fold change (log_2_FC) as the vertical axis and the names of the comparison groups as the horizontal axis, allowing for the simultaneous display of differentially expressed genes across multiple comparison groups. This method provides a detailed overview of the upregulated and downregulated genes within the comparison groups. As shown in Figure 4A, there are 952 genes that are upregulated and 2226 genes that are downregulated in the comparison between AP0 and AP9.

Based on the significantly different genes from each comparison group, we conducted a volcano plot analysis, as shown in Figure 4B. The volcano plot visually represents the differential gene expression between the comparison groups. Genes located closer to the ends of the plot exhibit greater differences. The horizontal axis indicates the log_2_ fold change between the two groups, while the vertical axis represents the −log_10_ values of the FDR or *p*-values for the differences between the two groups. Different colors denote upregulated and downregulated genes filtered according to specific thresholds, while black points represent genes with no significant differences.

Hierarchical clustering was performed on the differential gene expression patterns, and a heatmap was generated to present the clustering results. Genes with similar expression patterns may share common functions or participate in similar metabolic pathways and signaling pathways. Each gene was processed using z-scores, followed by the creation the diagram shown in Figure 4C. The variation in color gradient illustrates the expression levels of significantly different genes across various samples or groups, thereby revealing the patterns of gene expression changes among different samples or groups. Additionally, clustering analysis of samples and genes was conducted based on gene expression levels. Furthermore, to gain a deeper understanding of the process by which *A. pullulans* utilizes Huangjiu lees for the production of pullulan, we created a heatmap depicting the expression levels of genes involved in pullulan synthesis, as shown in Figure 4D. Genes such as *gel3*, *bxlB*, *GPH1*, and *MCA1587* exhibited increased expression levels in AP9, while *bglA* and *UGT80B1* showed elevated expression levels in AP0.

### 3.5. KEGG Enriched Pathway Analysis

**Figure 5:**
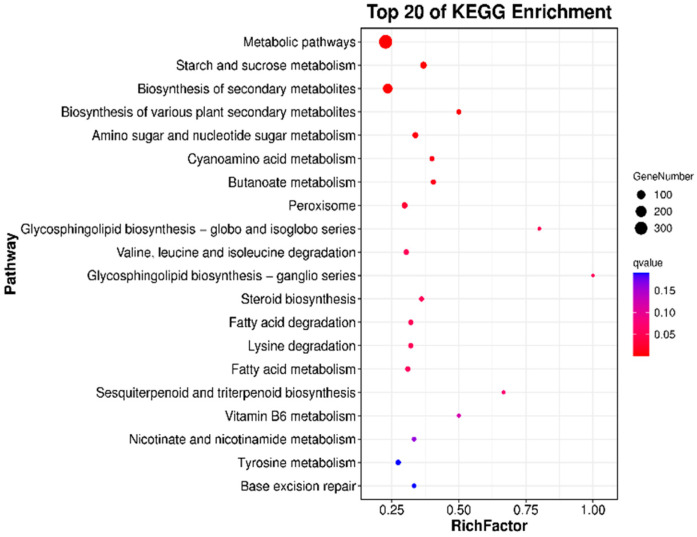
KEGG analysis of differentially expressed genes between AP0 and AP9.

The growth, development, and synthesis of microorganisms are coordinated by a complex network of metabolic pathways. Pathway enrichment analysis of differentially expressed genes can provide insights into the functions of these metabolic pathways and their interconnections. By mapping the transcripts of differentially expressed genes to the KEGG database and categorizing them through enrichment analysis, significant pathways that are enriched in differentially expressed genes compared to the overall genomic background can be identified using hypergeometric tests. A significance bubble plot can be employed to illustrate the proportion and significance of differentially expressed genes within each pathway category. In this plot, the size of the bubbles represents the number of differentially expressed genes enriched in the pathway, while the color indicates the significance of the enrichment. As shown in Figure 5, the comparison between the AP0 and AP9 groups reveals that the top 20 enriched pathways include metabolic pathways involving 349 differentially expressed genes; biosynthesis of secondary metabolites involving 147 differentially expressed genes; starch and sucrose metabolism involving 42 differentially expressed genes; and metabolism of amino sugars and nucleotide sugars involving 27 differentially expressed genes, among others. Additionally, pathways related to cyanuric acid metabolism; degradation of valine, leucine, and isoleucine; fatty acid metabolism; and tyrosine metabolism include over 55 differentially expressed genes. These analytical results indicate that significant differences in metabolic pathways and biosynthesis utilizing different carbon sources emerged in the primary strains after adaptive evolution experiments, highlighting the substantial impact of Huangjiu lees on the growth and development of *A. pullulans* and the biosynthesis of pullulan.

### 3.6. GO Enrichment Pathway Analysis

**Figure 6:**
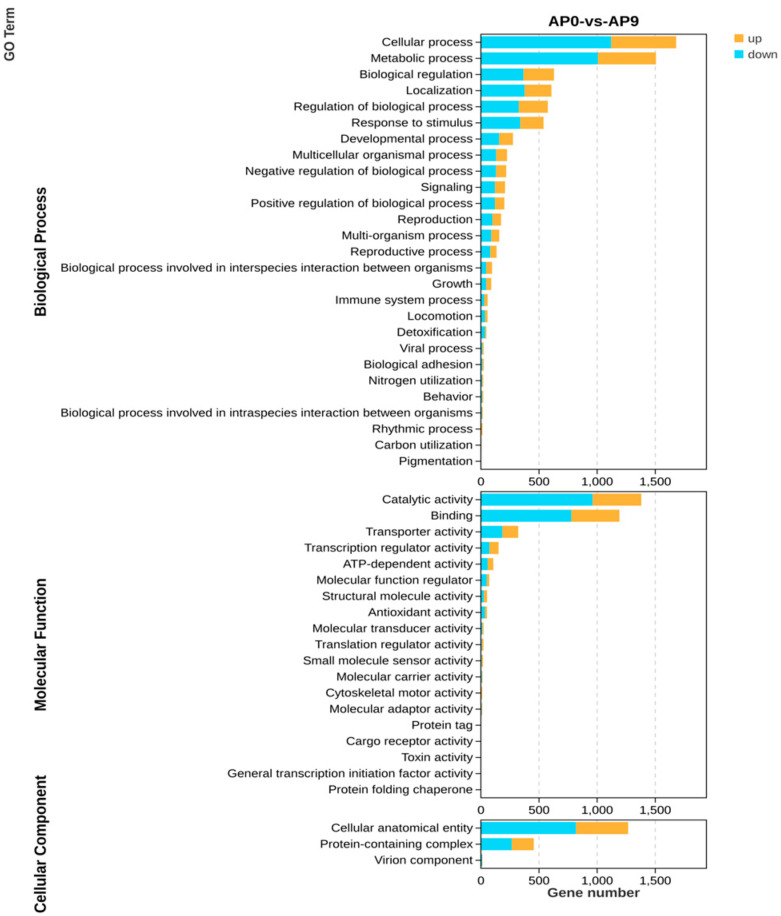
Biological processes, molecular functions, and cellular components of differentially expressed genes. In this figure, blue shows the number of down-regulated genes, while yellow shows the number of up-regulated genes.

The Gene Ontology (GO) database can be utilized to define and describe the functions of genes and proteins, as well as to classify genes based on the biological processes, cellular components, and molecular functions in which they are involved. Through significant enrichment analysis of GO functions, differences at the gene functional level between samples can be elucidated [31]. Analysis from Figure 6 indicates that differentially expressed genes primarily relate to biological processes such as cellular processes, metabolic processes, biological regulation, catalytic activity, and regulation of biological processes. When comparing the AP0 group with the AP9 group in terms of biological processes, it was found that the downregulated genes associated with cellular processes and metabolic processes (1121 and 1007, respectively) far exceeded the upregulated genes (599 and 499). Similarly, biological regulation, regulation of biological processes, and stimulus response processes exhibited a comparable number of upregulated and downregulated genes. In terms of molecular functions, the downregulated genes related to catalytic activity and binding (961 and 778) also significantly outnumbered the upregulated genes (419 and 414). For transport activities, transcription regulator activities, and ATP-dependent activities, a similar number of upregulated and downregulated genes were observed. Regarding cellular components, the downregulated genes associated with cellular anatomical entities (818) surpassed the upregulated genes (449), while the numbers of upregulated (190) and downregulated (265) genes related to protein complexes were roughly equivalent.

### 3.7. Validation of Key Expressed Genes

**Figure 7:**
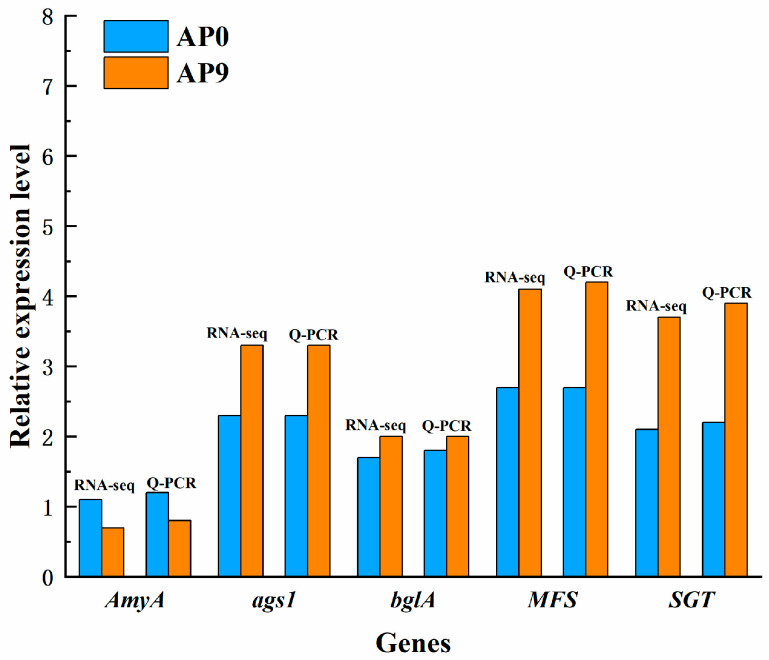
Validation of key genes.

Research indicates that α-amylase (amyA), α-1,3-glucan synthase (ags1), and β-glucosidase (bglA) are all involved in the synthesis of pullulan [32]. The Major Facilitator Superfamily (MFS) transporters, a special class of transmembrane transport proteins, participate in the transport of α-glucosides and other monosaccharides during the synthesis of pullulan. Therefore, MFS may play a crucial role as a transport protein in the pullulan synthesis process [33]. Transcriptomic data suggest that the high expression of the genes encoding AmyA, ags1, bglA, and MFS may be related to the synthesis and secretion of pullulan polysaccharides, a finding that has been validated by qPCR. Sterol glycosyltransferase (SGT) is one of the key enzymes in pullulan polysaccharide synthesis. Studies have shown that silencing the SGT gene results in reduced pullulan yield, indicating the enzyme’s active role in pullulan synthesis [34]. Transcriptomic data reveal that the expression level of the SGT gene in AP9 is higher than that in AP0, which also explains the previously observed higher pullulan yield in AP9 compared to AP0. The results of quantitative PCR analysis of five selected differential genes are consistent with the findings obtained from transcriptomic sequencing, confirming the reliability of the transcriptomic sequencing results (Figure 7).

### 3.8. Optimization of Fermentation Process of Evolved Strains

Differential gene expression analysis indicates that Huangjiu lees have significantly affected the metabolic pathways of the evolved strain AP9. Therefore, it is necessary to explore the optimal fermentation conditions to further enhance the yield of pullulan. This study employs a single-factor experimental design to analyze the effects of various factors, including fermentation time, inoculum quantity, temperature, initial pH, aeration rate, and rotational speed, on the yield and molecular weight of pullulan. A 5 L fermentation tank is utilized for scale-up production, optimizing the fermentation process of the evolved strain AP9 using Huangjiu lees as the substrate.

**Figure 8:**
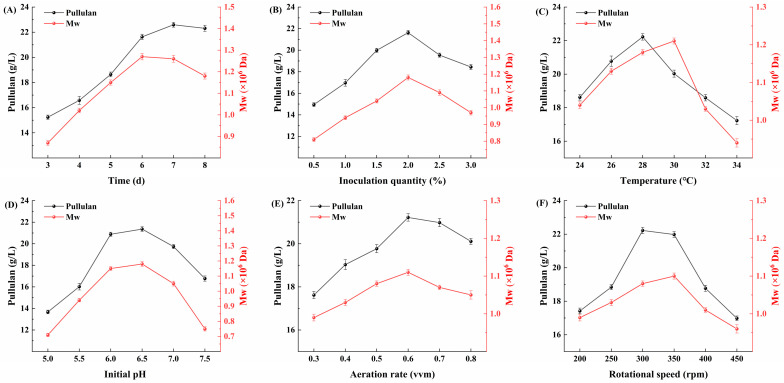
Single factor experiment. (**A**) Effect of time on fermentation; (**B**) effect of inoculum quantity on fermentation; (**C**) effect of temperature on fermentation; (**D**) effect of initial pH on fermentation; (**E**) effect of aeration rate on fermentation; (**F**) effect of rotational speed on fermentation.

Figure 8A indicates that fermentation time significantly affects the yield and molecular weight of pullulan. Within the range of 1 to 7 d, the yield of pullulan increases significantly with time, reaching its maximum at 7 d of fermentation. This suggests that the evolved strain AP9 is in a favorable growth state and can effectively utilize Huangjiu lees to produce pullulan. However, as the fermentation time extends to the 8th day, the yield of pullulan gradually stabilizes. In contrast, the molecular weight of pullulan reaches its peak at 6 d of fermentation, and it subsequently decreases significantly with further time. These results demonstrate that after a certain fermentation period, the yield of pullulan from the evolved strain AP9 stabilizes, but the degree of polymerization among pullulan molecules decreases. This phenomenon may be attributed to prolonged fermentation time leading to the cleavage of glycosidic bonds, resulting in a decrease in molecular weight. Although the highest yield of pullulan occurs at 7 d, a noticeable decline in molecular weight is already evident at that time. To ensure the quality of the pullulan product, a fermentation time of 6 d is therefore selected as the optimal duration.

From Figure 8B, it can be observed that when the inoculum quantity ranges from 0.5% to 2.0%, both the yield and molecular weight of pullulan show an increasing trend, reaching a maximum at 2.0%. However, as the inoculum quantity continues to increase beyond this point, both parameters exhibit a decline. These results indicate that within a certain range, an appropriate increase in inoculum quantity can accelerate the growth rate of the strain, shorten the fermentation time, facilitate the fermentation process, and enhance the accumulation of metabolic products, thereby improving production efficiency. Conversely, excessively high inoculum quantity may lead to rapid depletion of nutrients during the initial growth phase, resulting in restricted growth later on, which is detrimental to the fermentation process. Additionally, an excessively high inoculum can cause nutrient deficiencies in later stages, prompting microorganisms to utilize their own fermentation products for growth and reproduction, significantly reducing yield and further hindering product accumulation.

Research indicates that temperature is one of the most critical factors in the production of pullulan, with the optimal temperature for pullulan production generally ranging between 24 and 30 °C [35]. As shown in Figure 8C, the yield of pullulan peaks at 28 °C, while the molecular weight of pullulan reaches its maximum at 30 °C. Beyond these critical temperatures, both the yield and molecular weight decline rapidly with increasing temperature. This suggests that the evolved strain AP9 is sensitive to temperature, and elevated temperatures inhibit its growth. High temperatures may adversely affect the enzyme activity of the strain, disrupt growth processes, and even lead to the death of the strain. Thus, temperature plays a significant role in the fermentation process of *A. pullulans*.

Research has shown that *A. pullulans* exhibits a strong ability to adapt to various environmental conditions, thriving within a pH range of 3.0 to 8.5, with the highest pullulan yield occurring between pH 6.0 and 7.0 [36]. The experimental results of this study corroborate this finding, indicating that pH levels have varying degrees of impact on the growth of *A. pullulans* AP9. As illustrated in Figure 8D, both the pullulan yield and molecular weight are relatively low within the pH range of 5.0 to 5.5. The maximum pullulan yield and molecular weight are achieved at an initial pH of 6.5, with no significant differences observed between pH 6.0 and 6.5. Subsequently, both parameters gradually decline as the initial pH increases. These results suggest that *A. pullulans* is more suited for producing pullulan in slightly acidic environments, as both excessively acidic and alkaline conditions lead to a marked reduction in pullulan yield, likely due to the suppression of growth under these extreme pH conditions.

Dissolved oxygen is one of the most critical parameters in controlling aerobic fermentation. Due to the low solubility of oxygen in water, continuous aeration and agitation are necessary to meet the oxygen demands of various fermentation processes. The levels of dissolved oxygen significantly impact both the growth of microbial cells and the yield of the desired product [37]. The production of pullulan by *A. pullulans* occurs through an aerobic fermentation process, necessitating adequate oxygen supply to ensure the growth of the organism and the biosynthesis of pullulan [38]. As illustrated in Figure 8E, when the aeration rate is increased from 0.3 to 0.6 vvm, both the yield and molecular weight of pullulan increase with the aeration rate. However, further increases in the aeration rate lead to a decline in both pullulan yield and molecular weight, indicating that the optimal aeration rate for AP9 is 0.6 vvm. A rate exceeding 0.6 vvm results in decreased pullulan yield due to excessive aeration, which causes an oversaturation of dissolved oxygen in the fermentation tank. This excess oxygen can disrupt the cellular components of the microorganisms, leading to autolysis and ultimately reducing pullulan production.

As shown in Figure 8F, the highest yield of pullulan occurs at 300 rpm, while the molecular weight of pullulan is highest at 350 rpm. Insufficient rotational speed can lead to uneven dispersion of the culture, inadequate metabolic activity of the microorganisms, and prolonged growth cycles, which are detrimental to large-scale cultivation. In this study, we observed that when the rotational speed in the fermentation tank is below 250 rpm, the stirring blades are unable to effectively disperse the culture, resulting in the accumulation of Huangjiu lees at the bottom of the fermentation tank, which hinders the growth of *A. pullulans*. This is also the reason for the low pullulan yield at a rotational speed below 250 rpm. Conversely, excessively high rotational speed can generate shear forces that increase friction between cells, adversely affecting the normal growth of the strains and leading to a reduction in pullulan yield. Therefore, Lin et al. [39] suggest that stirred fermentation tanks are not suitable for the fermentation of *A. pullulans*, while air-lift fermentation tanks are more appropriate.

### 3.9. Optimization of Staged Fermentation Strategy

To shorten the fermentation time and further increase the yield of pullulan, this study employed a segmented fermentation process, which involves an initial fermentation with *Aspergillus niger* followed by fermentation with *A. pullulans* after sterilization. The purpose of this fermentation strategy is to utilize the cellulase produced by *Aspergillus niger* to degrade the cellulose in the Huangjiu lees into fermentable sugars that can be utilized by *A. pullulans*. This approach enables *A. pullulans* to better utilize the nutrients in the Huangjiu lees for pullulan production fermentation, thereby enhancing the substrate utilization efficiency of the Huangjiu lees.

**Figure 9:**
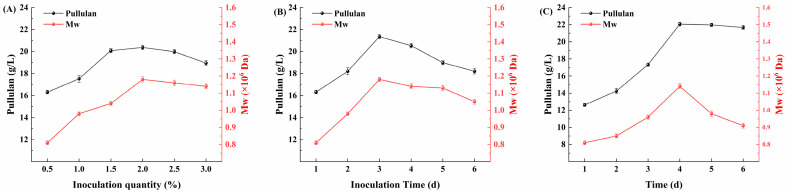
Segmented fermentation strategy. (**A**) Effect of *Aspergillus niger* inoculum quantity on fermentation; (**B**) effect of *Aspergillus niger* inoculation time on fermentation; (**C**) time course of pullulan production by *A. pullulans* AP9 using Huangjiu lees as substrate.

As shown in Figure 9A, the effect of different inoculum quantities of *Aspergillus niger* on the yield of pullulan was investigated. When the inoculum quantity ranged from 0.5% to 1.5%, both the yield and molecular weight of pullulan exhibited an increasing trend. However, when the inoculum quantity exceeded 2.0%, a gradual decline was observed. This reduction in pullulan yield may be attributed to the excessively high density of *Aspergillus niger* at elevated inoculum quantities, which leads to excessive acid production, resulting in an acidic environment that is detrimental to the fermentation process of *A. pullulans* in the later stages. Therefore, the optimal inoculation level of *Aspergillus niger* was determined to be 1.5%. Figure 9B explores the impact of inoculation time of *Aspergillus niger* on pullulan yield. As depicted, the highest yield and molecular weight of pullulan occurred at an inoculation time of 3 d, after which both parameters decreased with prolonged inoculation time. This decline may be due to the prolonged inoculation period causing *Aspergillus niger* to deplete excessive nutrients, thereby limiting the growth of *A. pullulans* in subsequent stages, which in turn leads to reduced pullulan yield. Consequently, the optimal inoculation time for *Aspergillus niger* was established as 3 d.

After optimizing the fermentation strategy for *A. pullulans*, a multi-strain segmented fermentation strategy utilizing *Aspergillus niger* and *A. pullulans* was employed. Using *A. pullulans* AP9, the production fermentation of pullulan was conducted with Huangjiu lees as the substrate. The specific cultivation conditions were as follows: a 1.5% inoculum quantity of *Aspergillus niger*, an inoculation time of 3 d, a 2% inoculum quantity of *A. pullulans*, a temperature of 28 °C, an initial pH of 6.5, an aeration rate of 0.6, and a rotational speed of 300 rpm. The fermentation time course for pullulan production by *A. pullulans* AP9 is illustrated in Figure 9C. It was observed that the maximum yield of pullulan reached 22.06 g/L with a molecular weight of 1.04 × 10^6^ Da at a fermentation duration of 4 d. This indicates that the multi-strain segmented fermentation strategy has shortened the fermentation cycle of *A. pullulans* while simultaneously increasing the yield of pullulan, which is of significant importance for the industrial scale-up of pullulan production.

## 4. Conclusions

In this study, we report the successful adaptive evolution of a strain of *Aureobasidium pullulan* AP9, which exhibits enhanced efficiency in utilizing Huangjiu lees for pullulan production. By optimizing fermentation conditions and employing a multi-strain staged fermentation strategy, we achieved a final pullulan yield of 22.06 g/L, with a molecular weight of 1.04 × 10^6^ Da, significantly surpassing the yield of 10.11 g/L produced by the original strain. Transcriptome analysis of *A. pullulans* reveals substantial alterations in the transcriptome of the evolved strains, resulting in distinct phenotypic variations. This provides a novel avenue for the genetic engineering of *A. pullulans*. Our findings not only support the industrial production of pullulan but also offer valuable insights into the resource utilization of Huangjiu lees.

## Figures and Tables

**Table 1:** Parameter of fermentation conditions.

Single Factor	Variable
Fermentation time (d)	3, 4, 5, 6, 7, 8
Initial pH	5.0, 5.5, 6.0, 6.5, 7.0, 7.5
Inoculation quantity (%)	0.5, 1.0, 1.5, 2.0, 2.5, 3.0
Aeration rate (vvm)	0.3, 0.4, 0.5, 0.6, 0.7, 0.8
Rotational speed (rpm)	200, 250, 300, 350, 400, 450
Temperature (°C)	24, 26, 28, 30, 32, 34

**Table 2:** Parameters of staged fermentation experiment.

Influencing Factors	Variable Range	Other Parameters
Aspergillus niger inoculation quantity (%)	1, 2, 3, 4, 5, 6	pH 6.5, 5 d, 28 °C, 300 rpm, 0.6 vvm
Aspergillus niger inoculation time (d)	0.5, 1.0, 1.5, 2.0, 2.5, 3.0	pH 6.5, 1.0% inoculation quantity, 28 °C, 300 rpm, 0.6 vvm

## Data Availability

The data presented in this study are available on request from the corresponding author.

## Supplementary Materials

The following supporting information can be downloaded at https://www.mdpi.com/article/10.3390/foods13233874/s1: Table S1: Primers used in this study; Table S2: Number of genes detected in each sample.

## References

[1] Hu Y., Pan L., Dun Y., Peng N., Liang Y., Zhao S. (2014). Conversion of yellow wine lees into high-protein yeast culture by solid-state fermentation. Biotechnol. Biotechnol. Equip..

[2] Yao K.Y., Wei Z.H., Xie Y.Y., Wang D.M., Liu H.Y., Fang D., Ma M.R., Liu J.X. (2020). Lactation performance and nitrogen utilization of dairy cows on diets including unfermented or fermented yellow wine lees mix. Livest. Sci..

[3] Fabian H.T., Catalina G.E. (2022). Characterization and chemical modification of pullulan produced from a submerged culture of *Aureobasidium pullulans* ATCC 15233. Polym. Test..

[4] Guo J., Reis J., Salze G., Rhodes M., Tilton S., Davis D.A. (2019). Using high protein distiller’s dried grain product to replace corn protein concentrate and fishmeal in practical diets for the Pacific white shrimp Litopenaeus vannamei. J. World Aquac. Soc..

[5] Yao K., Xia Q., Cao Y., Chen B., Cai J., Liu C. (2023). Feeding effect of yellow wine lees fermented with Candida utilis and Bacillus subtilis in the cow diet on milk composition. Food Biosci..

[6] Li C., Kong D., Yao X., Ma X., Wei C., Wang H. (2022). Resource Recycling Utilization of Distillers Grains for Preparing Cationic Quaternary Ammonium—Ammonium Material and Adsorption of Acid Yellow 11. Sustainability.

[7] Sharma N., Prasad G.S., Choudhury A.R. (2013). Utilization of corn steep liquor for biosynthesis of pullulan, an important exopolysaccharide. Carbohydr. Polym..

[8] Xia Z., Wu S., Pan S. (2011). Effect of two-stage controlled pH and temperature on pullulan production by *Auerobasidium pullulans*. Carbohydr. Polym..

[9] Duan X., Chi Z., Wang L., Wang X. (2008). Influence of different sugars on pullulan production and activities of α-phosphoglucose mutase, UDPG-pyrophosphorylase and glucosyltransferase involved in pullulan synthesis in *Aureobasidium pullulans* Y68. Carbohydr. Polym..

[10] Shingel K.I. (2004). Current knowledge on biosynthesis, biological activity, and chemical modification of the exopolysaccharide, pullulan. Carbohydr. Res..

[11] West T.P. (2024). Impact of Aureobasidium Species Strain Improvement on the Production of the Polysaccharide Pullulan. Polysaccharides.

[12] Kang C., Hao L., Zhang L., Zheng Z., Yang Y. (2018). Isolation, purification and antioxidant activity of polysaccharides from the leaves of maca (*Lepidium meyenii*). Int. J. Biol. Macromol..

[13] Wang W., Zhang K., Lin C., Zhao S., Guan J., Zhou W., Ru X., Cong H., Yang Q. (2023). Influence of Cmr1 in the Regulation of Antioxidant Function Melanin Biosynthesis in *Aureobasidium pullulans*. Foods.

[14] Chen L., Ji F., Bao Y., Xia J., Guo L., Wang J., Li Y. (2017). Biocompatible cationic pullulan-g-desoxycholic acid-g-PEI micelles used to co-deliver drug and gene for cancer therapy. Mater. Sci. Eng. C.

[15] Mayra S., Pau T., Amparo C. (2019). Improving Functional Properties of Cassava Starch-Based Films by Incorporating Xanthan, Gellan, or Pullulan Gums. Int. J. Polym. Sci..

[16] María C.M., Fernandes A.F.A., Leda d.A.G., Pereira S.V., Aline P.C., Nayeli O., José C.M., Franco M.P.R., César S.J. (2023). Production and applications of pullulan from lignocellulosic biomass: Challenges and perspectives. Bioresour. Technol..

[17] Cheng K.C., Demirci A., Catchmark J.M. (2011). Pullulan: Biosynthesis, production, and applications. Appl. Microbiol. Biotechnol..

[18] Wang D., Ju X., Zhou D., Wei G. (2014). Efficient production of pullulan using rice hull hydrolysate by adaptive laboratory evolution of *Aureobasidium pullulans*. Bioresour. Technol..

[19] Herrera A., Téllez-Luis S.J., Ramıírez J.A., Vázquez M. (2003). Production of Xylose from Sorghum Straw Using Hydrochloric Acid. J. Cereal Sci..

[20] Lavarack B.P., Griffin G.J., Rodman D. (2002). The acid hydrolysis of sugarcane bagasse hemicellulose to produce xylose, arabinose, glucose and other products. Biomass Bioenergy.

[21] Lazaridou A., Roukas T., Biliaderis C.G., Vaikousi H. (2002). Characterization of pullulan produced from beet molasses by *Aureobasidium pullulans* in a stirred tank reactor under varying agitation. Enzym. Microb. Technol..

[22] Tagne R.F.T., Santos M.M.C., Antunes F.A.F., Shibukawa V.P., Miano S.B., Kenfack J.A.A., Silva S.S.d., Ngomade S.B.L., Santos J.C. (2024). Pullulan Production from Sugarcane Bagasse Hemicellulosic Hydrolysate by *Aureobasidium pullulans* ATCC 42023 inBubble Column Reactor. Fermentation.

[23] Zhang K., Wang W., Yang Q. (2023). Transcriptome Analysis Reveals the Regulation of *Aureobasidium pullulans* under Different pH Stress. Int. J. Mol. Sci..

[24] Zhang K., Lin C., Zhao S., Wang W., Zhou W., Ru X., Cong H., Yang Q. (2023). The role of pH transcription factor Appacc in upregulation of pullulan biosynthesis in *Aureobasidium pullulans* using potato waste as a substrate. Int. J. Biol. Macromol..

[25] Wang Q., Liang L., Xi F., Tian G., Mao Q., Meng X. (2020). Adsorption of Azo Dye Acid Red 73 onto Rice Wine Lees: Adsorption Kinetics and Isotherms. Adv. Mater. Sci. Eng..

[26] He C., Zhang X., Zhang Z., Wang C., Wang D., Wei G. (2022). Whole-crop biorefinery of corn biomass for pullulan production by *Aureobasidium pullulans*. Bioresour. Technol..

[27] Liu N.-N., Chi Z., Wang Q.-Q., Hong J., Liu G.-L., Hu Z., Chi Z.-M. (2017). Simultaneous production of both high molecular weight pullulan and oligosaccharides by *Aureobasdium melanogenum* P16 isolated from a mangrove ecosystem. Int. J. Biol. Macromol..

[28] Cheng K.-C. (2011). Evaluation of Medium Composition and Fermentation Parameters on Pullulan Production by *Aureobasidium pullulans*. Food Sci. Technol. Int..

[29] Jiang B., Liu J., Wang J., Zhao G., Zhao Z. (2024). Adaptive Evolution for the Efficient Production of High-Quality d-Lactic Acid Using Engineered Klebsiella pneumoniae. Microorganisms.

[30] Zhang Y., Feng J., Wang P., Xia J., Li X., Zou X. (2019). CRISPR/Cas9-mediated efficient genome editing via protoplast-based transformation in yeast-like fungus *Aureobasidium pullulans*. Gene.

[31] Gostinčar C., Ohm R.A., Kogej T., Sonjak S., Turk M., Zajc J., Zalar P., Grube M., Sun H., Han J. (2014). Genome sequencing of four *Aureobasidium pullulans* varieties: Biotechnological potential, stress tolerance, and description of new species. BMC Genom..

[32] Liu N.N., Chi Z., Liu G.L., Chen T.J., Jiang H., Hu Z., Chi Z.M. (2018). α-Amylase, glucoamylase and isopullulanase determine molecular weight of pullulan produced by *Aureobasidium melanogenum* P16. Int. J. Biol. Macromol..

[33] Ma L., Chen L., Zhang L., Zou G., Liu R., Jiang Y., Zhou Z. (2016). RNA Sequencing Reveals Xyr1 as a Transcription Factor Regulating Gene Expression beyond Carbohydrate Metabolism. BioMed Res. Int..

[34] Chen T.-J., Liu G.-L., Chen L., Yang G., Hu Z., Chi Z.-M., Chi Z. (2020). Alternative primers are required for pullulan biosynthesis in *Aureobasidium melanogenum* P16. Int. J. Biol. Macromol..

[35] Aquinas N., Chithra C.H., Bhat M.R. (2024). Progress in bioproduction, characterization and applications of pullulan: A review. Polym. Bull..

[36] Wani S.M., Mir S.A., Khanday F.A., Masoodi F.A. (2021). Advances in pullulan production from agro-based wastes by *Aureobasidium pullulans* and its applications. Innov. Food Sci. Emerg. Technol..

[37] Xu Y., Ye D., Zhang W., Wang Y., Li J., Zhang L., Huang J., Zhu X., Liao Q. (2024). Dual-scale pore network modeling of two-phase transport in anode porous transport layer and catalyst layer of proton exchange membrane electrolyzers. Energy Convers. Manag..

[38] Shu L., Yang M., Zhao H., Li T., Yang L., Zou X., Li Y. (2019). Process optimization in a stirred tank bioreactor based on CFD-Taguchi method: A case study. J. Clean. Prod..

[39] Lin C., Zhang K., Zhao S., Wang W., Ru X., Song J., Cong H., Yang Q. (2022). Screening and identification of a strain of Aureobasidium pullulans and its application in potato starch industrial waste. Environ. Res..

