# Supplementary material for: Investigation of Efficient Pullulan Synthesis Utilizing Huangjiu Lees as a Substrate
Source: Foods. 2024 Nov 29;13(23):3874. doi: 10.3390/foods13233874 (PMC11640504; doi:10.3390/foods13233874)
Supplement: Supplementary file 1 [file foods-13-03874-s001.zip › foods-3316990-supplementary.pdf]

foods

Supplementary Materials

Table S1. Primers used in this study

Gene ID	Name	Sequence (5′ to 3′)
AmyA	AmyAF	ACTGATCTCTTGTCCTGCACTTC
	AmyAR	AATCTCGAGGTTGGGTAGAGAAC
ags1	ags1F	CTAGAATGGACGATATGGACAGC
	ags1R	GACAAGCAGACAGGAATGACTCT
bglA	bglAF	CAGCTACTCTGACATTTCGGTCT
	bglAR	GGTAGAGTTGAGGAAGGGGTAGA
MFS	MFSF	GAGTACAGGGCACTTGCATTTAG
	MFSR	GTCCATAAGTCTCCTTGACGATG
SGT	SGTF	GAGACATCGTTAGCAGCTGAAGT
	SGTR	CTTGATAGTCCAGACTGCTGGTT

Table S2. Number of genes detected in each sample

Sample Name	Knou Gene Num
AP0-1	13439(92.91%)
AP0-2	13473(93.15%)
AP0-3	13436(92.86%)
AP9-1	13257(91.66%)
AP9-2	13224(91.43%)
AP9-3	13226(91.44%)
